# Imaging in gynecological disease (28): clinical and ultrasound characteristics of serous and mucinous cystadenomas in the adnexa

**DOI:** 10.1002/uog.29248

**Published:** 2025-05-26

**Authors:** F. Mascilini, F. Moro, T. Pasciuto, P. Sladkevicius, W. Froyman, L. Jokubkiene, C. Van Holsbeke, D. Franchi, E. Epstein, S. Guerriero, V. Chiappa, F. Buonomo, M. J. Kudla, J. L. Alcázar, L. Hochberg, F. Ciccarone, L. Quagliozzi, G. Scambia, D. Timmerman, L. Valentin, A. C. Testa

**Affiliations:** ^1^ Dipartimento Scienze della Salute della Donna, del Bambino e di Sanità Pubblica Fondazione Policlinico Universitario Agostino Gemelli, IRCCS Rome Italy; ^2^ UniCamillus International Medical University Rome Italy; ^3^ Research Core Facility Data Collection G‐STEP Fondazione Policlinico Universitario Agostino Gemelli IRCCS Rome Italy; ^4^ Section of Hygiene, University Department of Life Sciences and Public Health Università Cattolica Del Sacro Cuore Rome Italy; ^5^ Department of Obstetrics and Gynecology Skåne University Hospital Malmö Sweden; ^6^ Department of Clinical Sciences Malmö Lund University Lund Sweden; ^7^ Department of Obstetrics and Gynecology University Hospital Leuven Leuven Belgium; ^8^ Department of Development and Regeneration KU Leuven Leuven Belgium; ^9^ Department of Obstetrics and Gynecology Ziekenhuis Oost‐Limburg Genk Belgium; ^10^ Preventive Gynecology Unit, Division of Gynecology European Institute of Oncology IRCCS Milan Italy; ^11^ Department of Clinical Science and Education Södersjukhuset, Karolinska Institutet Stockholm Sweden; ^12^ Department of Obstetrics and Gynecology University of Cagliari, Policlinico Universitario Duilio Casula Cagliari Italy; ^13^ Department of Gynecologic Oncology Fondazione IRCCS Istituto Nazionale dei Tumori Milan Italy; ^14^ Institute for Maternal and Child Health IRCCS ‘Burlo Garofolo’ Trieste Italy; ^15^ Department of Perinatology and Oncological Gynecology, Faculty of Medical Sciences Medical University of Silesia Katowice Poland; ^16^ Department of Obstetrics and Gynecology Clinica Universidad de Navarra, School of Medicine Pamplona Spain; ^17^ Imaged‐Based Gynecology Service, Department of Obstetrics and Gynecology, University of South Florida Morsani College of Medicine Tampa FL USA; ^18^ Dipartimento Universitario Scienze della Vita e Sanità Pubblica Università Cattolica del Sacro Cuore Rome Italy

**Keywords:** cystadenoma, ovarian neoplasm, ovary, ultrasonography

## Abstract

**Objective:**

To describe the clinical and ultrasound characteristics of serous and mucinous cystadenomas in the adnexa.

**Methods:**

This was a retrospective international multicenter study. Using the International Ovarian Tumor Analysis (IOTA) database, patients with a histological diagnosis of serous or mucinous cystadenoma who had undergone preoperative ultrasound examination between 1999 and 2016 (IOTA studies phase 1, 1b, 2, 3 and 5) were identified. All masses were described using the standardized IOTA terminology. The diagnosis assigned by the original ultrasound examiner based on subjective assessment was recorded. Two reviewers assessed the available digital ultrasound images using pattern recognition to identify typical sonographic features of cystadenomas.

**Results:**

A total of 1318 patients were included: 687 (52.1%) with serous cystadenomas and 631 (47.9%) with mucinous cystadenomas. Based on the data recorded prospectively in the IOTA database, for serous cystadenomas the median diameter of the largest tumor was 68 (range, 14–320) mm. Most serous cystadenomas were described as unilateral (588/687 (85.6%)), with unilocular (274/687 (39.9%)) or multilocular (221/687 (32.2%)) morphology, and most had anechoic cyst content (508/687 (73.9%)). Most serous cystadenomas were not vascularized (color score of 1; 327/687 (47.6%)) or were poorly vascularized (color score of  2; 253/687 (36.8%)) on color Doppler examination. The original ultrasound examiner correctly classified 91.1% (626/687) of serous cystadenomas as benign and suggested the correct specific diagnosis in 51.5% (354/687) of tumors. For mucinous cystadenomas, the median diameter of the largest tumor was 93 (range, 12–550) mm. Most mucinous cystadenomas were described as unilateral (594/631 (94.1%)) with multilocular morphology (357/631 (56.6%)), and most manifested low‐level echogenicity (334/631 (52.9%)). Most mucinous cystadenomas were poorly (color score of 2; 248/631 (39.3%)) or moderately (color score of 3; 194/631 (30.7%)) vascularized on color Doppler examination. The original ultrasound examiner correctly classified 87.5% (552/631) of mucinous cystadenomas as benign and suggested the correct specific diagnosis in 42.9% (271/631) of tumors. Based on pattern recognition (review of ultrasound images available for 433 tumors), the most typical sonographic features of serous cystadenomas were unilocular cyst (100/211 (47.4%)) or multilocular cyst with < 10 cyst locules (71/211 (33.6%)), whereas the typical features of mucinous cystadenomas were multilocular cyst with < 10 cyst locules (99/222 (44.6%)), unilocular cyst (78/222 (35.1%)) or multilocular cyst with > 10 cyst locules (31/222 (14.0%)). A honeycomb nodule was found in some mucinous cystadenomas (31/222 (14.0%)) but was not found in serous cystadenomas.

**Conclusions:**

Serous and mucinous cystadenomas exhibit typical sonographic features, allowing ultrasound examiners to assign a correct specific diagnosis to most tumors. Recognizing the ultrasound features of cystadenomas and avoiding misdiagnosing them as malignant can help prevent surgery for these benign tumors in asymptomatic patients. © 2025 The Author(s). *Ultrasound in Obstetrics & Gynecology* published by John Wiley & Sons Ltd on behalf of International Society of Ultrasound in Obstetrics and Gynecology.

## INTRODUCTION

The aim of this study was to describe the clinical and ultrasound characteristics of serous and mucinous cystadenomas in the adnexa, in order to enhance ultrasound knowledge of the histological subtypes of ovarian tumors to improve preoperative diagnosis and optimize management.

According to the 2020 World Health Organization (WHO) classification of tumors of the ovary^1^, serous cystadenomas, serous cystadenofibromas and serous surface papillomas are benign serous tumors composed of cells resembling Fallopian tube epithelium. Serous papillary cystadenomas were once included as a subgroup of benign serous tumors^2^, but they are now included in the serous cystadenofibroma group^3^. Benign serous tumors are common, accounting for two‐thirds of benign ovarian epithelial tumors^3^. Benign serous tumors are diagnosed in women of all ages, with reported mean age at diagnosis ranging from 40 to 60 years^3^.

Mucinous cystadenomas are defined by WHO as benign tumors with gastrointestinal or Müllerian‐type mucinous epithelium^1^. Benign mucinous cystadenomas account for 13% of all benign ovarian epithelial neoplasms^3^ and 80% of all primary mucinous ovarian tumors. Mucinous cystadenomas are diagnosed most commonly in women aged 30–70 years, with a mean age at diagnosis of 50 years^1^.

The epithelial lining of serous cystadenomas consists of non‐stratified cuboidal or columnar cells (resembling tubal secretory or ciliated cells) in varying proportions. In some tumors, the epithelial lining is flattened and nondescript. Serous cystadenomas are predominantly cystic^1^. If < 10% of the tumor volume (taking into account cyst walls, septa and solid components) shows epithelial proliferation when viewed by microscopy, the lesion is classified as serous cystadenoma with focal epithelial proliferation, which is considered a benign condition, even though data are limited. If > 10% of the total tumor volume shows epithelial proliferation, the tumor is classified as a serous borderline tumor^1^.

Mucinous cystadenomas are composed of multiple cysts and glands lined by simple non‐stratified mucinous epithelium, which resembles Müllerian, gastric foveolar‐type or intestinal epithelium containing goblet cells, and sometimes neuroendocrine or Paneth cells^1^. Rare papillae may be seen^1^.

On occasion, cystadenomas have endocervical‐type mucinous epithelium. These are referred to as Müllerian‐type mucinous, endocervical‐like mucinous or seromucinous cystadenomas. The epithelium closely resembles endocervical mucinous epithelium, with columnar cells having apical mucin and basally situated bland nuclei^3^. They often exhibit a papillary architecture, in contrast to the purely glandular pattern seen in the mucinous type.

Serous cystadenomas vary widely in size. They are typically unilocular, but may be multilocular, and exhibit smooth outer and inner surfaces. They are bilateral in 10–20% of cases^3^. Benign serous tumors are composed of cysts filled with clear watery fluid or thin mucoid material. Occasionally, they contain thicker mucus‐like material, more typical of mucinous neoplasms^3^.

Mucinous cystadenomas are typically unilateral (95%) and multilocular, with a smooth outer surface. They range in size from a few centimeters to > 30 cm (mean, 10 cm)^3^. They may contain thin or thick mucinous fluid^3^. Sometimes mural nodules are seen^1^.

Seromucinous cystadenomas are unilocular or multilocular cystic tumors. This subtype is very rare^3^.

The symptoms and signs associated with cystadenomas are non‐specific. The most common symptoms are abdominal or pelvic pain and symptoms related to an abdominal pelvic mass. Small lesions are typically incidental findings^1^. Rarely, patients with mucinous cystadenomas present with estrogenic or androgenic manifestations, secondary to stromal luteinization^1^. Serous and mucinous cystadenomas are benign. However, recurrence of serous cystadenomas may be seen after incomplete excision. Furthermore, recurrence of mucinous cystadenomas may be seen after cystectomy or after rupture or spillage of cyst contents^1^.

## METHODS

This was a retrospective international multicenter study. The study was approved by the Ethics Committee of Fondazione Policlinico Universitario Agostino Gemelli, IRCCS, Rome, Italy (N15197/23). Using the International Ovarian Tumor Analysis (IOTA) database, we identified patients with a histological diagnosis of serous or mucinous cystadenoma who had undergone preoperative ultrasound examination between 1999 and 2016 (IOTA studies phase 1, 1b, 2, 3 and 5)^4^, ^5^, ^6^, ^7^, ^8^, ^9^. The IOTA database is a research database that includes all patients prospectively enrolled in multicenter IOTA studies. It contains clinical, ultrasound and histological data of patients with an adnexal mass examined with ultrasound imaging before surgery. Thirty‐one ultrasound centers contributed patient data to the study (Table S1). All clinical and ultrasound characteristics of the study population were retrieved from the IOTA database. Written or oral consent was obtained from all patients or from a guardian of minors. All patients were examined using transvaginal ultrasound imaging (supplemented with a transabdominal scan if necessary) using a standardized examination technique and following a strict research protocol, and all masses were described using the standardized IOTA terminology^10^. The ultrasound examiners were European Federation of Societies for Ultrasound in Medicine and Biology (EFSUMB) Level II or Level III examiners^11^. The ultrasound examinations were carried out using high‐end ultrasound equipment. The frequency range was 5.0–9.0 MHz for vaginal probes and 3.5–5.0 MHz for abdominal probes.

For patients with bilateral masses with the same histology, we used data from the dominant mass in the statistical analysis. The dominant mass was the one with the most complex ultrasound appearance; if both masses had similar ultrasound morphology, the dominant mass was considered to be the largest mass or the mass that was most easily accessible on ultrasound. Using IOTA terminology, a papillary projection was defined as a projection of solid tissue into a cyst cavity, with a height of at least 3 mm^10^. Papillary projections differ from other solid components in that they protrude into the cyst cavity, while other solid components do not. The following tumor measurements are described in the study: largest diameter of the largest solid component, height of the largest papillary projection and the largest diameter of the mass. The results of Doppler ultrasound examination are reported in terms of color score^10^, as follows: 1 = no blood flow detected in the tumor; 2 = minimal flow detected; 3 = moderate flow detected; 4 = abundant color (highly vascular with marked blood flow). Results based on subjective assessment (benign or malignant tumor, specific histological diagnosis) by the original ultrasound examiner and recorded in the IOTA database were also analyzed.

The principal investigator at each participating center was responsible for retrieving the digital images for each patient included in the study. The investigators uploaded the available digital images into REDCap, an electronic data capture tool hosted at Fondazione Policlinico Universitario Agostino Gemelli IRCCS^12^.

Two EFSUMB Level III examiners (F.Ma. and F.Mo.) reviewed the ultrasound images using pattern recognition^13^ to identify any typical sonographic characteristics. This included searching specifically for the presence of a ‘honeycomb nodule’, which is defined as a multilocular nodule arising from the inner cyst wall^14^. Their consensus opinion was used in the statistical analysis for pattern recognition. Disagreements between reviewers were resolved through discussion, with a third reviewer available for unresolved cases (A.C.T.). The minimum image quality requirements were that the lesion and external contours of the tumor were clearly visible, and the image resolution was sufficient to see details.

Pseudoanonymized data were managed according to European regulations on privacy of data (UE2016/679).

### Statistical analysis

Results are presented as *n* (%) for categorical variables and as median (range) or mean ± SD for continuous variables, as appropriate. The Shapiro–Wilk test was used to test data distribution for normality. The statistical analysis was performed by a biostatistician (T.P.) using Stata/BE version 17.0 (StataCorp LP, College Station, TX, USA). The tumors were classified as serous or mucinous cystadenomas based on the histology provided by the pathologist at each participating center. Tumors classified as seromucinous cystadenomas by the pathologist were included in the mucinous group because of their rarity and because they have been described previously as mucinous tumors in pathology textbooks^3^.

## RESULTS

**Figure 1:**
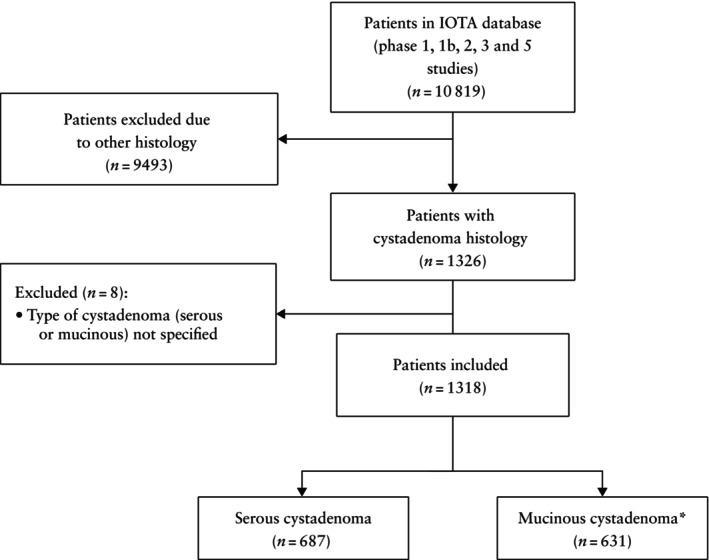
Flowchart showing included patients with histological diagnosis of serous or mucinous cystadenoma between 1999 and 2016 identified from the International Ovarian Tumor Analysis (IOTA) database. *Includes four seromucinous cystadenomas.

From the IOTA database (IOTA studies phases 1, 1b, 2, 3 and 5)^4^, ^5^, ^6^, ^7^, ^8^, ^9^ we identified 1326 patients with histological confirmation of ovarian cystadenoma. The type of cystadenoma (serous or mucinous) was not specified in eight patients and these were excluded. A total of 1318 patients were included in the analysis: 687 (52.1%) with serous cystadenomas and 631 (47.9%) with mucinous cystadenomas, four of which were seromucinous cystadenomas (Figure 1). Twenty‐three of the 631 mucinous cystadenomas were included in a published single‐center study describing the clinical and ultrasound features of mucinous tumors^15^.

Clinical characteristics of the patients are given in Table 1 and ultrasound characteristics of the tumors are given in Table 2.

**Table 1:** Clinical characteristics of 1318 patients with serous or mucinous cystadenoma identified from the International Ovarian Tumor Analysis (IOTA) database

Characteristic	Total (*n* = 1318)	Serous cystadenoma (*n* = 687)	Mucinous cystadenoma (*n* = 631)
History of ovarian cancer			
Family	33/1314 (2.5)	25/684 (3.7)	8/630 (1.3)
Personal	9/1315 (0.7)	4/684 (0.6)	5/631 (0.8)
Age (years)	51.5 ± 16.1; 52 (13–92)	53.7 ± 15.8; 54 (15–92)	49.1 ± 16.1; 50 (13–90)
Postmenopausal	678/1312 (51.7)	388/684 (56.7)	290/628 (46.2)
Nulliparous	303/995 (30.5)	156/527 (29.6)	147/468 (31.4)
CA125 (U/mL)*	15 (1–13 510)	14 (1–6438)	15 (2–13 510)
IOTA study phase			
1	189 (14.3)	97 (14.1)	92 (14.6)
1b	63 (4.8)	26 (3.8)	37 (5.9)
2	285 (21.6)	153 (22.3)	132 (20.9)
3	323 (24.5)	160 (23.3)	163 (25.8)
5	458 (34.7)	251 (36.5)	207 (32.8)

Data are given as *n*/*N* (%), mean ± SD or median (range). Denominators are included for missing data.

* Information available for 870/1318 (66.0%) cases.

**Table 2:** Ultrasound characteristics and diagnosis based on subjective assessment by original ultrasound examiner for serous and mucinous cystadenomas identified from the International Ovarian Tumor Analysis (IOTA) database

Characteristic	Total (*n* = 1318)	Serous cystadenoma (*n* = 687)	Mucinous cystadenoma (*n* = 631)
Laterality			
Bilateral masses	136 (10.3)	99 (14.4)	37 (5.9)
Morphological features			
Largest diameter of tumor (mm)	77 (12–550)	68 (14–320)	93 (12–550)
Type of mass			
Unilocular	431 (32.7)	274 (39.9)	157 (24.9)
Multilocular	578 (43.9)	221 (32.2)	357 (56.6)
Unilocular‐solid	99 (7.5)	83 (12.1)	16 (2.5)
Multilocular‐solid	206 (15.6)	105 (15.3)	101 (16.0)
Solid	4 (0.3)	4 (0.6)	0 (0)
Number of locules for multilocular and multilocular‐solid masses with ≤ 10 locules	4 (2–10)	4 (2–10)	5 (2–10)
> 10 locules	79/784 (10.1)	24/326 (7.4)	55/458 (12.0)
Echogenicity of cyst fluid			
Anechoic	701 (53.2)	508 (73.9)	193 (30.6)
Low‐level	473 (35.9)	139 (20.2)	334 (52.9)
Ground‐glass	61 (4.6)	18 (2.6)	43 (6.8)
Hemorrhagic	6 (0.5)	2 (0.3)	4 (0.6)
Mixed	73 (5.5)	16 (2.3)	57 (9.0)
No cyst fluid	4 (0.3)	4 (0.6)	0 (0)
Largest diameter of largest solid component (mm)	12 (1–97)	11 (1–97)	16 (1–74)
Papillary projection(s)	206/1315 (15.7)	143/684 (20.9)	63 (10.0)
Number of papillary projections			
1	132/206 (64.1)	91/143 (63.6)	41/63 (65.1)
2	30/206 (14.6)	23/143 (16.1)	7/63 (11.1)
3	17/206 (8.3)	8/143 (5.6)	9/63 (14.3)
> 3	27/206 (13.1)	21/143 (14.7)	6/63 (9.5)
Height of largest papillary projection (mm)	6 (3–45)	6 (3–28)	7 (3–45)
Flow in papillary projection, if present	65/206 (31.6)	48/143 (33.6)	17/63 (27.0)
Shadowing	82 (6.2)	45 (6.6)	37 (5.9)
Other features			
Ovarian crescent sign	267/965 (27.7)	160/501 (31.9)	107/464 (23.1)
Ascites	17 (1.3)	5 (0.7)	12 (1.9)
Fluid in pouch of Douglas	159 (12.1)	61 (8.9)	98 (15.5)
Depth of fluid in pouch of Douglas (mm)	10 (1–54)	9 (1–37)	12 (1–54)
Color score			
1 (no flow)	488 (37.0)	327 (47.6)	161 (25.5)
2	501 (38.0)	253 (36.8)	248 (39.3)
3	296 (22.5)	102 (14.8)	194 (30.7)
4 (abundant flow)	33 (2.5)	5 (0.7)	28 (4.4)
Subjective assessment			
Diagnosis based on subjective assessment			
Benign	1178 (89.4)	626 (91.1)	552 (87.5)
Borderline or malignant	140 (10.6)	61 (8.9)	79 (12.5)
Specific diagnosis			
Serous cystadenoma/cystadenofibroma	482 (36.6)	354 (51.5)	128 (20.3)
Mucinous cystadenoma/cystadenofibroma	345 (26.2)	74 (10.8)	271 (42.9)
Simple cyst/paraovarian cyst/parasalpingeal cyst	115 (8.7)	94 (13.7)	21 (3.3)
Endometrioma	32 (2.4)	12 (1.7)	20 (3.2)
Dermoid	29 (2.2)	8 (1.2)	21 (3.3)
Cystadenoma/cystadenofibroma (not specified)	24 (1.8)	13 (1.9)	11 (1.7)
Functional ovarian cyst	13 (1.0)	8 (1.2)	5 (0.8)
Hydrosalpinx/chronic PID	12 (0.9)	7 (1.0)	5 (0.8)
Peritoneal pseudocyst	6 (0.5)	6 (0.9)	0 (0)
Abscess/salpingitis/PID	5 (0.4)	0 (0)	5 (0.8)
Fibroma/fibrothecoma/thecofibroma	6 (0.5)	5 (0.7)	1 (0.2)
Benign rare tumor	3 (0.2)	3 (0.4)	0 (0)
Hydrosalpinx or cystadenoma	1 (0.1)	1 (0.1)	0 (0)
Borderline tumor	94 (7.1)	38 (5.5)	56 (8.9)
Primary invasive tumor	24 (1.8)	11 (1.6)	13 (2.1)
Metastases to the ovary	5 (0.4)	2 (0.3)	3 (0.5)
Not possible	59 (4.5)	25 (3.6)	34 (5.4)
Not performed (IOTA 1b)	63 (4.8)	26 (3.8)	37 (5.9)

Data are given as *n*/*N* (%) or median (range). PID, pelvic inflammatory disease.

### Serous cystadenomas

The median age of patients with serous tumors was 54 (range, 15–92) years, and most were postmenopausal (388/684 (56.7%)). According to the data recorded prospectively in the IOTA database, the median diameter of the largest tumor measured on ultrasound was 68 (range, 14–320) mm. Most serous cystadenomas were unilateral (588/687 (85.6%)), most had unilocular (274/687 (39.9%)) or multilocular (221/687 (32.2%)) morphology and most had anechoic cyst content (508/687 (73.9%)). Papillary projections were described in 143/684 (20.9%) tumors. The median height of the largest papillary projection was 6 (range, 3–28) mm, and in most cases the papillary projections were not vascularized. Most serous cystadenomas were not vascularized (color score of 1, 327/687 (47.6%)) or were poorly vascularized (color score of 2, 253/687 (36.8%)) on color Doppler examination. The original ultrasound examiner correctly classified 91.1% (626/687) of the serous cystadenomas as benign and suggested the correct specific diagnosis in 51.5% (354/687) of the tumors. Clinical and ultrasound characteristics of serous cystadenomas that were classified correctly as benign and those that were misdiagnosed as malignant (61/687 (8.9%)) by the original examiner are reported in Tables S2 and S3, respectively; examples of misclassified tumors are presented in Figure S1. As reported in Table S3, tumors misdiagnosed as malignant were more often bilateral than those that were classified correctly. Serous cystadenomas misdiagnosed as malignant were classified most frequently as unilocular‐solid or multilocular‐solid and most were vascularized on color Doppler imaging. Those classified correctly as benign were more often unilocular or multilocular cysts, often with no detectable vascularization. Papillary projections were reported more frequently in the misdiagnosed group.

**Figure 2:**
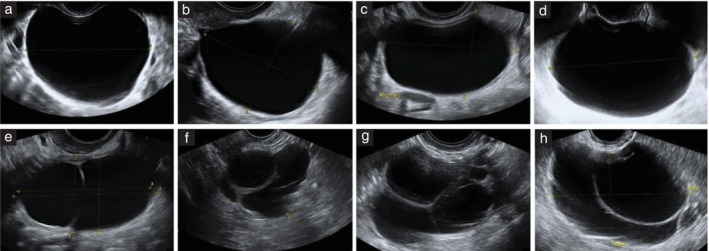
Grayscale ultrasound images showing typical features of serous cystadenomas. (a–d) Unilocular anechoic cysts with smooth walls. (e–h) Multilocular cysts showing thin septations and < 10 cyst locules.

**Figure 3:**
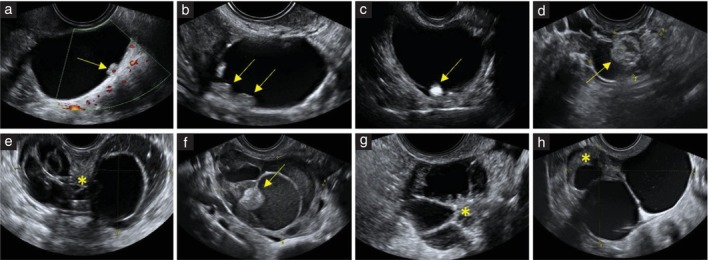
Power Doppler (a) and grayscale (b–h) ultrasound images showing less common features of serous cystadenomas. (a–c) Unilocular‐solid cysts with papillary projections (arrows). (d–h) Multilocular‐solid cysts with papillary projections (arrows) or other solid components (asterisks).

Digital ultrasound images were available for 211 serous cystadenomas: only grayscale images for 88 (41.7%) tumors, only color or power Doppler images for 3 (1.4%) tumors and both grayscale and color or power Doppler images for 120 (56.9%) tumors. All images were good quality (lesion and external contours of the tumor clearly visible and image resolution sufficient to see details in the image). Based on the available ultrasound images and using pattern recognition, the most typical sonographic features of serous cystadenomas were a unilocular cyst (100/211 (47.4%)) or a multilocular cyst with < 10 cyst locules (71/211 (33.6%)) (Figure 2). Less common features of serous cystadenomas were a unilocular‐solid cyst with papillary projections (24/211 (11.4%)), multilocular cyst with > 10 cyst locules (4/211 (1.9%)), multilocular‐solid cyst with papillary projections or with small solid components that were not papillary projections (11/211 (5.2%)), or a solid tumor (1/211 (0.5%)) (Figure 3).

### Mucinous cystadenomas

The median age of patients with mucinous tumors was 50 (range, 13–90) years, and most patients were premenopausal (338/628 (53.8%)). According to the data recorded prospectively in the IOTA database, at ultrasound examination most mucinous cystadenomas were described as unilateral lesions (594/631 (94.1%)), most were multilocular cysts (357/631 (56.6%)), most manifested low‐level echogenicity (334/631 (52.9%)) and, on average, they were larger than serous cystadenomas. Papillary projections were reported in 63/631 (10.0%) tumors. Most mucinous cystadenomas were poorly (color score of  2, 248/631 (39.3%)) or moderately (color score of 3, 194/631 (30.7%)) vascularized on color Doppler examination. The original ultrasound examiner correctly classified 87.5% (552/631) of the mucinous cystadenomas as benign and suggested the correct specific diagnosis in 42.9% (271/631) of tumors. Clinical and ultrasound characteristics of mucinous cystadenomas classified correctly as benign and those misdiagnosed as malignant (79/631 (12.5%)) by the original examiner are reported in Tables S2 and S3, respectively; examples of misclassified tumors are shown in Figure S2. Misdiagnosed tumors were larger than those classified correctly as benign. In addition, they were more frequently multilocular‐solid and contained > 10 cyst locules compared with mucinous cystadenomas that had been classified correctly (Table S3).

**Figure 4:**
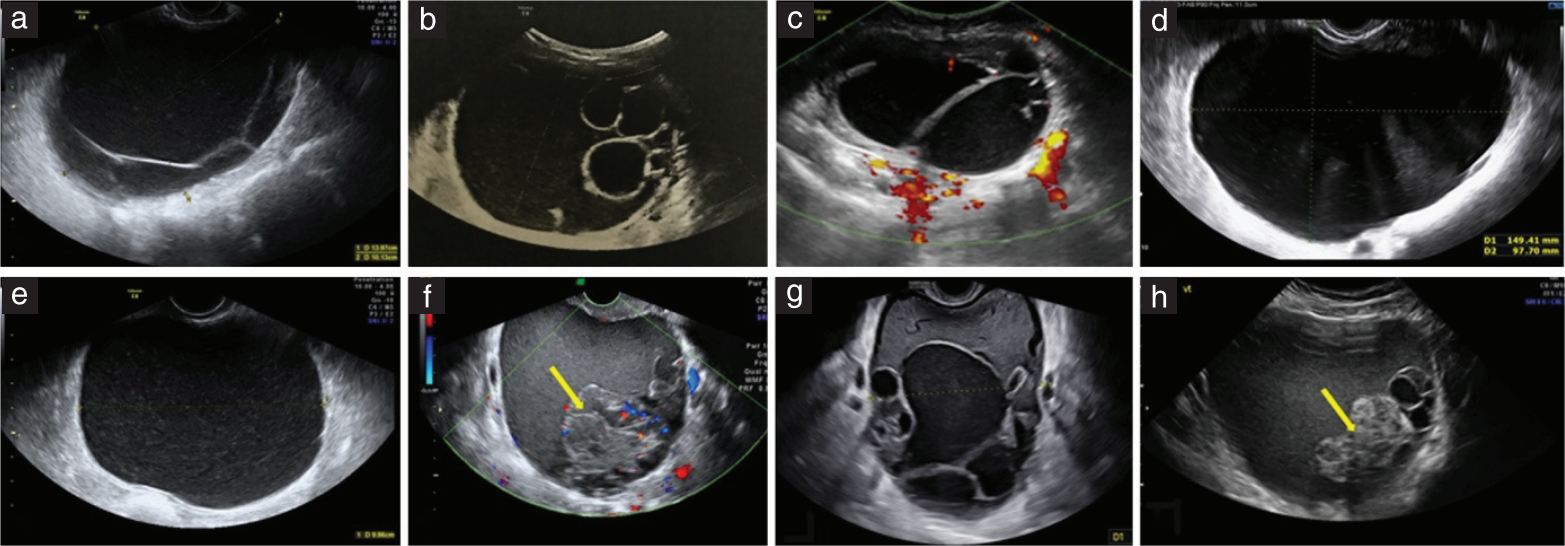
Grayscale (a,b,d,e,g,h), power (c) and color Doppler (f) ultrasound images showing typical features of mucinous cystadenomas. Most mucinous cystadenomas were multilocular cysts with < 10 locules (a–c), unilocular cysts (d,e) or multilocular cysts with > 10 locules (f–h). A honeycomb nodule can be seen in (f) and (h) (arrows).

**Figure 5:**
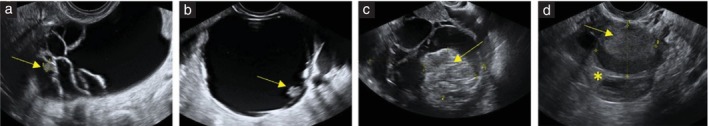
Grayscale ultrasound images showing less common features of mucinous cystadenomas. (a–c) Multilocular‐solid cyst (a), unilocular‐solid cyst (b) and multilocular‐solid cyst (c) with papillary projections (arrows). (d) Multilocular‐solid cyst with papillary projections (arrow) and a large solid component (asterisk).

Digital ultrasound images were available for 222 patients with mucinous cystadenomas: only grayscale images for 72 (32.4%) tumors, only color or power Doppler images for one (0.5%) tumor and both grayscale and color or power Doppler images for 149 (67.1%) tumors. All images were good quality (lesion and external contours of the tumor clearly visible and image resolution sufficient to see details in the image). Based on the available ultrasound images and using pattern recognition, the most typical sonographic features of mucinous cystadenomas were: multilocular cyst with < 10 cyst locules (99/222 (44.6%)), unilocular cyst (78/222 (35.1%)) or multilocular cyst with > 10 cyst locules (31/222 (14.0%)) (Figure 4). Less common sonographic features of mucinous cystadenomas were a multilocular‐solid cyst with papillary projections (7/222 (3.2%)), unilocular‐solid cyst with papillary projections (6/222 (2.7%)) or multilocular‐solid cyst with large solid components but no papillary projections (1/222 (0.5%)) (Figure 5). A honeycomb nodule was seen in 31/222 (14.0%) mucinous cystadenomas (Figure S2).

## DISCUSSION

To the best of our knowledge, this is the largest study describing the ultrasound characteristics of serous and mucinous cystadenomas. Most serous cystadenomas were unilocular cysts, whereas most mucinous tumors were multilocular cysts. Most serous cysts contained anechoic cyst fluid, while most mucinous cysts contained cyst fluid of low‐level echogenicity. Mucinous cysts were larger than serous cysts, contained a higher number of cyst locules and demonstrated greater vascularization than serous cysts.

Our ultrasound results are consistent with the macroscopic appearance of serous and mucinous cystadenomas described in pathology textbooks, in that the mucinous tumors were larger and more often multilocular than the serous tumors, and because their cyst contents were most often of low‐level echogenicity (corresponding to mucinous cyst contents), whereas the contents of serous tumors was most often anechoic (corresponding to watery cyst contents)^1^, ^2^, ^3^.

The major strength of our study is the high number of tumor cases included. However, the retrospective analysis and the lack of digital images for a high proportion of patients (885/1318 (67.1%)) is a limitation. The latter may have affected the results of pattern recognition. Moreover, the two examiners who reviewed the images were not blinded to the histopathological diagnosis, and this may have influenced their interpretations. Another limitation is that we did not examine interobserver agreement for pattern recognition. Instead, the results of pattern recognition were the consensus opinion of the two reviewers of the images.

Our results are in accordance with those of previous studies reporting the ultrasound characteristics of cystadenomas. These studies were based on a small series of cases and were conducted before 2020, highlighting a gap in the literature. In 1983, Moyle *et al*.^16^ described the ultrasound features of 16 mucinous and 25 serous cystadenomas, reporting that the two types of tumor had a similar appearance on ultrasound: anechoic cysts with no solid components or containing small (< 5% of the whole cyst) amounts of echogenic tissue. In a single‐center study describing the ultrasound features of 57 benign mucinous cystadenomas, the typical ultrasound feature was a multilocular cyst with > 10 locules^15^. In that study, a honeycomb nodule was observed in 7.0% (4/57) compared with 14.0% in the present study. The largest series of cystadenomas (405 patients) was published in 2022 by Suh‐Burgmann *et al*.^17^ The authors described the growth rate of serous cystadenomas (histologically confirmed) during ultrasound follow‐up, but they did not describe the ultrasound characteristics of the tumors. The median time interval between first detection of the mass on ultrasound examination and surgery was 1.3 (range, 0–22) years, and the median annual growth rate based on first and last measurements was 0.83 cm/year for mucinous tumors and 0.51 cm/year for serous tumors.

A subset of the cystadenomas in our series exhibited ultrasound characteristics that were similar to those of serous cystadenofibromas^18^ in that they manifested papillary projections or other solid components. This could be explained by a change in pathology nomenclature and classification over the years. According to Mutter and Prat^2^, there are four types of serous tumors: serous cystadenoma, serous cystadenofibroma, serous surface papilloma and serous papillary cystadenoma. The WHO classification^1^ includes only three types of serous benign tumors: serous cystadenoma, serous cystadenofibroma and serous surface papilloma. Therefore, serous papillary cystadenomas are now classified as cystadenofibromas. We hypothesize that some serous papillary cystadenomas may have been classified by the pathologist as cystadenomas in our series. The presence of papillary projections in cystadenomas may cause difficulties in discriminating between a cystadenoma and a serous borderline ovarian tumor. However, papillary projections of serous borderline tumors typically have an irregular surface and are vascularized on color or power Doppler examination^19^, whereas papillary projections in benign serous tumors (i.e. cystadenofibromas) tend to have shadowing without detectable blood flow^18^, ^20^.

The differential diagnosis between benign, borderline and invasive mucinous tumors is challenging because of the overlapping ultrasound (and macroscopic) features between the three categories. We show that many benign mucinous cystadenomas are multilocular cysts with > 10 locules and many exhibit low‐level echogenicity of cyst fluid. Benign mucinous cystadenomas share these features with mucinous borderline tumors (Figure S2)^15^. The honeycomb nodule has been described as a pathognomonic feature of borderline mucinous tumors and was observed in 53.3% of mucinous borderline tumors by Yazbek *et al*.^14^ and in 26% by Moro *et al*.^15^ However, in our series, a honeycomb nodule was found in 14.0% of benign mucinous cystadenomas. It may be that the honeycomb nodule is a marker of a mucinous tumor rather than a specific sign of borderline mucinous tumor.

Our study has potential clinical implications. Assigning a correct diagnosis of cystadenoma can help triage patients with ovarian masses more effectively. For instance, asymptomatic patients with cysts, classified as benign cystadenomas, can be managed conservatively with clinical and ultrasound follow‐up^8^. In cases in which the diagnosis based on subjective assessment is uncertain, using the IOTA Assessment of Different NEoplasias in the adneXa (ADNEX) model, published in 2014^21^, could be considered. ADNEX has high accuracy in distinguishing benign from malignant adnexal masses and also provides the likelihood of a tumor being borderline, Stage I primary invasive, Stage II–IV primary invasive or metastatic^9^. ADNEX was not applied in our study population because many patients were evaluated before the development of the ADNEX model. It is therefore unclear whether ADNEX would have performed better than subjective assessment in those serous and mucinous cystadenomas that were misclassified as malignant by the original ultrasound examiner.

In conclusion, serous and mucinous cystadenomas manifest typical sonographic features, allowing ultrasound examiners to assign a correct diagnosis in most cases. Our results, and those of other publications in the Imaging in Gynecological Disease series^15^, ^18^, ^19^, ^22^, ^23^, ^24^, ^25^, ^26^, ^27^, ^28^, ^29^, ^30^, ^31^, ^32^, ^33^, ^34^, ^35^, ^36^, ^37^, ^38^, ^39^, establish the foundation for prospective studies estimating the ability of ultrasound examiners to assign a correct specific diagnosis to adnexal masses using pattern recognition. It remains to be seen whether IOTA models such as ADNEX, or use of radiomics or other artificial intelligence algorithms, can improve discrimination between benign, borderline and malignant serous and mucinous tumors.

## Supporting information


**Figure S1** Grayscale (a,b,c,e) and color Doppler (d) ultrasound images of benign serous cystadenomas misclassified by original ultrasound examiner as malignant (borderline) ovarian tumors. (a,b) Multilocular‐solid cysts with solid components. (c,e) Unilocular‐solid cysts with papillary projections. (d) Multilocular cysts with < 10 locules.

**Figure S2** Grayscale ultrasound images of benign mucinous cystadenomas misclassified as borderline ovarian tumors (a–e) or primary invasive tumors (f–h). (a,b,d,e,d,g,h) Multilocular cysts with > 10 locules. (c,f) Multilocular‐solid cysts with > 10 than locules. The overlapping features with benign mucinous cystadenomas are clear. Honeycomb nodule is seen (yellow arrows).

**Table S1** List of 31 participating centers and number of patients, per center, with histological diagnosis of serous or mucinous cystadenoma (*n* = 1318), identified from the International Ovarian Tumor Analysis database
**Tables S2 and S3** Clinical characteristics (Table S2) and ultrasound characteristics (Table S3) of 1318 women with serous or mucinous cystadenoma correctly classified or misdiagnosed as borderline tumor or invasive malignancy by original ultrasound examiner

## Data Availability

The data that support the findings of this study are available from the corresponding author upon reasonable request.
