# Supplementary figures and images for: Imaging in gynecological disease (28): clinical and ultrasound characteristics of serous and mucinous cystadenomas in the adnexa
Source: Ultrasound Obstet Gynecol. 2025 May 26;66(2):233–41. doi: 10.1002/uog.29248 (PMC12317306; doi:10.1002/uog.29248)

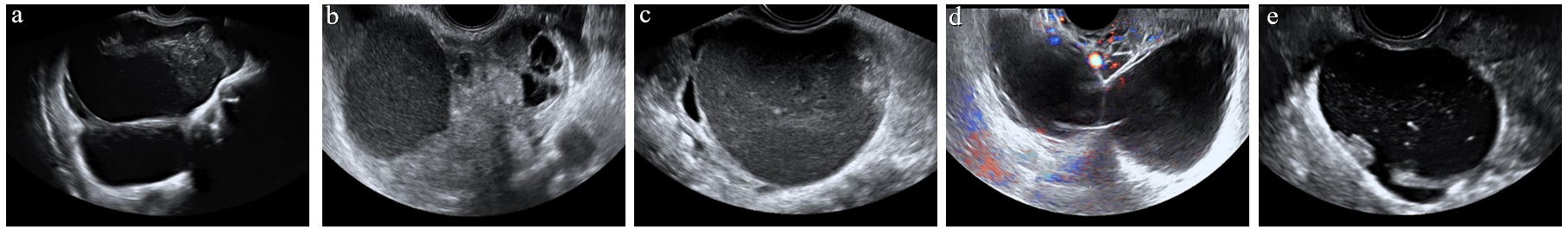

Supplement: Supplementary file 1 — Figure S1 Grayscale (a,b,c,e) and color Doppler (d) ultrasound images of benign serous cystadenomas misclassified by original ultrasound examiner as malignant (borderline) ovarian tumors. (a,b) Multilocular‐solid cysts with solid components. (c,e) Unilocular‐solid cysts with papillary projections. (d) Multilocular cysts with < 10 locules. [file UOG-66-233-s001.png]

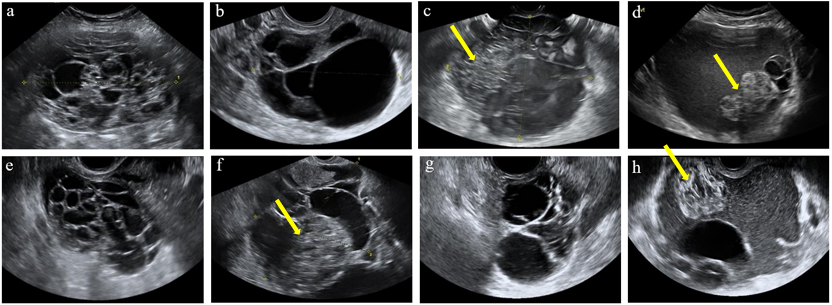

Supplement: Supplementary file 2 — Figure S2 Grayscale ultrasound images of benign mucinous cystadenomas misclassified as borderline ovarian tumors (a–e) or primary invasive tumors (f–h). (a,b,d,e,d,g,h) Multilocular cysts with > 10 locules. (c,f) Multilocular‐solid cysts with > 10 than locules. The overlapping features with benign mucinous cystadenomas are clear. Honeycomb nodule is seen (yellow arrows). [file UOG-66-233-s002.png]
