# Supplementary material for: Imaging in gynecological disease (28): clinical and ultrasound characteristics of serous and mucinous cystadenomas in the adnexa
Source: Ultrasound Obstet Gynecol. 2025 May 26;66(2):233–41. doi: 10.1002/uog.29248 (PMC12317306; doi:10.1002/uog.29248)
Supplement: Supplementary file 3 — Table S1 List of 31 participating centers and number of patients, per center, with histological diagnosis of serous or mucinous cystadenoma (n = 1318), identified from the International Ovarian Tumor Analysis database Tables S2 and S3 Clinical characteristics (Table S2) and ultrasound characteristics (Table S3) of 1318 women with serous or mucinous cystadenoma correctly classified or misdiagnosed as borderline tumor or invasive malignancy by original ultrasound examiner [file UOG-66-233-s003.docx]

| **Table S1** List of 31 participating centers and number of patients, per center, with histological diagnosis of serous or mucinous cystadenoma (n=1318), identified from the International Ovarian Tumor Analysis database. | | |
| --- | --- | --- |
| Center | Patients included in the study (n=1318) | Patients with at least one image available for pattern recognition (n=459) |
| Sweden, Malmo, Skåne University Hospital | 240 | 122 |
| Belgium, Leuven, Universitaire Ziekenhuizen Leuven | 153 | 61 |
| Belgium, Genk, Ziekenhuis Oost-Limburg | 122 | 102 |
| Italy, Rome, Fondazione Policlinico Universitario A. Gemelli, IRCCS | 120 | 46 |
| Italy, Monza, San Gerardo Hospital | 98 | 0 |
| Italy, Milan, DCS Sacco University of Milan | 77 | 0 |
| Italy, Milan, Istituto Europeo di Oncologia | 64 | 40 |
| Sweden, Stockholm, Södersjukhuset | 56 | 29 |
| Italy, Cagliari, Ospedale San Giovanni di Dio | 50 | 40 |
| Italy, Bologna, Sant'Orsola Malpighi Hospital | 47 | 0 |
| Greece, Athens, Alexandra Maternity Hospital | 44 | 0 |
| Czech Republic, Prague, Charles University | 30 | 0 |
| Poland, Lublin, Medical University of Lublin | 30 | 0 |
| France, Paris, Hôpital Boucicaut | 23 | 0 |
| United Kingdom, London, University College Hospital | 20 | 0 |
| Italy, Trieste, IRCCS Burlo Garofolo | 17 | 13 |
| Italy, Udine, Azienda Sanitaria Universitaria Integrata di Udine | 17 | 0 |
| France, Le Chesnay, Centre Européen de Diagnostic et d’Exploration de la Femme | 16 | 0 |
| Italy, Naples, Università degli Studi di Napoli | 16 | 0 |
| Sweden, Lund, Skåne University Hospital | 15 | 0 |
| Spain, Barcelona, Institut Universitari Dexeus, | 14 | 0 |
| Poland, Katowice, Central University Hospital | 10 | 3 |
| Spain, Pamplona, University of Navarra | 7 | 0 |
| Italy, Florence, University of Florence | 6 | 0 |
| China, Beijing, Chinese PLA General Hospital | 5 | 0 |
| Italy, Milan, Macedonio Melloni Hospital | 5 | 0 |
| Italy, Milan, IRCCS National Cancer Institute | 4 | 3 |
| Canada, Ontario, McMaster University, St. Joseph’s Hospital | 4 | 0 |
| Italy, Rome, Sapienza University Hospital | 3 | 0 |
| United Kingdom, London, Queen Charlotte's and Chelsea Hospital | 3 | 0 |
| Italy, Milan, Ospedale dei Bambini Vittore Buzzi | 2 | 0 |
|  |  |  |

| **Table S2** Clinical characteristics of 1318 women with serous or mucinous cystadenoma correctly classified or misdiagnosed as borderline tumor or invasive malignancy by original ultrasound examiner. | | | | | |
| --- | --- | --- | --- | --- | --- |
| Characteristic | Serous | |  | Mucinous | |
|  | Not misdiagnosed  N=626 | Misdiagnosed N=61 |  | Not misdiagnosed  N=552 | Misdiagnosed N=79 |
| History of ovarian cancer |  |  |  |  |  |
| Family | 22/624 (3.5) | 3/60 (5.0) |  | 7/551 (1.3) | 1/79 (1.3) |
| Personal | 2/623 (0.3) | 2/61 (3.3) |  | 1/552 (0.2) | 4/79 (5.1) |
| Age, years | 53.9 ± 15.8 | 51.5 ± 15.4 |  | 48.9 ± 16.2 | 50.9 ± 15.6 |
| Median (min-max) | 54.5 (15-92) | 53 (18-79) |  | 49 (13-90) | 52 (20-88) |
| Postmenopause | 355/623 (57.0) | 33/61 (54.1) |  | 251/549 (45.7) | 39/79 (49.4) |
| Nulliparous | 142/487 (29.2) | 14/40 (35.0) |  | 131/418 (31.3) | 16/50 (32.0) |
| CA125, U/mL ‡ | 14 (1-6438) | 17.5 (6-154) |  | 15 (2-553) | 26 (9-13510) |
| IOTA phase |  |  |  |  |  |
| 1 | 89/626 (14.2) | 8/61 (13.1) |  | 85/552 (15.4) | 7/79 (8.9) |
| 1 b | 23/626 (3.7) | 3/61 (4.9) |  | 34/552 (6.2) | 3/79 (3.8) |
| 2 | 143/626 (22.8) | 10/61 (16.4) |  | 120/552 (21.7) | 12/79 (15.2) |
| 3 | 139/626 (22.2) | 21/61 (34.4) |  | 134/552 (24.3) | 29/79 (36.7) |
| 5 | 232/626 (37.1) | 19/61 (31.1) |  | 179/552 (32.4) | 28/79 (35.4) |
| Results are given as n/N (%) for categorical variables and as median (min-max) and/or mean±standard deviation for continuous characteristics as appropriate. For categorical variables, denominators differ due to missing data. ‡ Information available for 870/1318 (60.0%) cases. | | | | | |

| **Table S3** Ultrasound characteristics of 1318 women with serous and mucinous cystadenoma correctly classified or misdiagnosed as borderline tumor or invasive malignancy by original ultrasound examiner. | | | | | |
| --- | --- | --- | --- | --- | --- |
| Characteristic | Serous | |  | Mucinous | |
|  | Not misdiagnosed  N=626 | Misdiagnosed N=61 |  | Not misdiagnosed  N=552 | Misdiagnosed N=79 |
| *Laterality* |  |  |  |  |  |
| Bilateral masses | 85/626 (13.6) | 14/61 (23.0) |  | 33/552 (6.0) | 4/79 (5.1) |
| *Morphological features* |  |  |  |  |  |
| Largest diameter of lesion, mm | 68 (15-320) | 60 (14-300) |  | 88 (12-550) | 153 (16-450) |
| Type of mass |  |  |  |  |  |
| Unilocular | 273/626 (43.6) | 1/61 (1.6) |  | 156/552 (28.3) | 1/79 (1.3) |
| Multilocular | 212/626 (33.9) | 9/61 (14.8) |  | 324/552 (58.7) | 33/79 (41.8) |
| Unilocular solid | 56/626 (8.9) | 27/61 (44.3) |  | 10/552 (1.8) | 6/79 (7.6) |
| Multilocular solid | 82/626 (13.1) | 23/61 (37.7) |  | 62/552 (11.2) | 39/79 (49.4) |
| Solid | 3/626 (0.5) | 1/61 (1.6) |  | 0/552 (0.0) | 0/79 (0.0) |
| Number of locules for multilocular and mutilocular solid masses | 4 (2-10) | 4 (2-10) |  | 5 (2-10) | 6 (2-9) |
| More than 10 locules | 18/294 (6.1) | 6/32 (18.8) |  | 34/386 (8.8) | 21/72 (29.2) |
| Echogenicity of cyst fluid |  |  |  |  |  |
| Anechoic | 480/626 (76.7) | 28/61 (45.9) |  | 172/552 (31.2) | 21/79 (26.6) |
| Low-level | 116/626 (18.5) | 23/61 (37.7) |  | 294/552 (53.3) | 40/79 (50.6) |
| Ground glass | 14/626 (2.2) | 4/61 (6.6) |  | 35/552 (6.3) | 8/79 (10.1) |
| Hemorrhagic | 2/626 (0.3) | 0/61 (0.0) |  | 2/552 (0.4) | 2/79 (2.5) |
| Mixed | 11/626 (1.8) | 5/61 (8.2) |  | 49/552 (8.9) | 8/79 (10.1) |
| No cyst fluid | 3/626 (0.5) | 1/61 (1.6) |  | 0/552 (0.0) | 0/79 (0.0) |
| Largest diameter of largest solid component, mm | 9 (1-96) | 14 (1-97) |  | 14 (1-57) | 24 (1-74) |
| Papillary projection(s) | 105/625 (16.8) | 38/59 (64.4) |  | 37/552 (6.7) | 26/79 (32.9) |
| Number of papillary projections |  |  |  |  |  |
| 1 | 72/105 (68.6) | 19/38 (50.0) |  | 25/37 (67.6) | 16/26 (61.5) |
| 2 | 14/105 (13.3) | 9/38 (23.7) |  | 3/37 (8.1) | 4/26 (15.4) |
| 3 | 8/105 (7.6) | 0/38 (0.0) |  | 6/37 (16.2) | 3/26 (11.5) |
| >3 | 11/105 (10.5) | 10/38 (26.3) |  | 3/37 (8.1) | 3/26 (11.5) |
| Height of largest papillary projection, mm | 5 (3-23) | 8 (3-28) |  | 6 (3-45) | 9 (3-36) |
| Flow in papillary projection, if papillary projection present | 22/105 (21.0) | 26/38 (68.4) |  | 4/37 (10.8) | 13/26 (50.0) |
| Shadowing | 44/626 (7.0) | 1/61 (1.6) |  | 33/552 (6.0) | 4/79 (5.1) |
| *Other features* |  |  |  |  |  |
| Ovarian crescent sign | 145/454 (31.9) | 15/47 (31.9) |  | 101/397 (25.4) | 6/67 (9.0) |
| Ascites | 2/626 (0.3) | 3/61 (4.9) |  | 6/552 (1.1) | 6/79 (7.6) |
| Fluid in pouch of Douglas | 47/626 (7.5) | 14/61 (23.0) |  | 78/552 (14.1) | 20/79 (25.3) |
| Fluid in pouch of Douglas, mm | 8 (1-37) | 14 (2-35) |  | 9 (1-46) | 16.5 (3-54) |
| *Color Doppler results* |  |  |  |  |  |
| Color score |  |  |  |  |  |
| 1 | 319/626 (51.0) | 8/61 (13.1) |  | 149/552 (27.0) | 12/79 (15.2) |
| 2 | 224/626 (35.8) | 29/61 (47.5) |  | 219/552 (39.7) | 29/79 (36.7) |
| 3 | 82/626 (13.1) | 20/61 (32.8) |  | 168/552 (30.4) | 26/79 (32.9) |
| 4 | 1/626 (0.2) | 4/61 (6.6) |  | 16/552 (2.9) | 12/79 (15.2) |
| *Subjective assessment* |  |  |  |  |  |
| Diagnosis based on subjective assessment |  |  |  |  |  |
| Benign | 626/626 (100.0) | 0/61 (0.0) |  | 552/552 (100.0) | 0/79 (0.0) |
| Borderline or malignant | 0/626 (0.0) | 61/61 (100.0) |  | 0/552 (0.0) | 79/79 (100.0) |
| Specific diagnosis |  |  |  |  |  |
| Serous cystademoma/cyadenofibroma | 354/626 (56.5) | 0/61 (0.0) |  | 128/552 (23.2) | 0/79 (0.0) |
| Mucinous cystademoma/cyadenofibroma | 74/626 (11.8) | 0/61 (0.0) |  | 271/552 (49.1) | 0/79 (0.0) |
| Simple cyst / paraovarian cyst / parasalpingeal cyst | 94/626 (15.0) | 0/61 (0.0) |  | 21/552 (3.8) | 0/79 (0.0) |
| Endometrioma | 12/626 (1.9) | 0/61 (0.0) |  | 20/552 (3.6) | 0/79 (0.0) |
| Dermoid | 8/626 (1.3) | 0/61 (0.0) |  | 21/552 (3.8) | 0/79 (0.0) |
| Cystadenoma | 13/626 (2.1) | 0/61 (0.0) |  | 11/552 (2.0) | 0/79 (0.0) |
| Functional ovarian cyst | 8/626 (1.3) | 0/61 (0.0) |  | 5/552 (0.9) | 0/79 (0.0) |
| Hydrosalpinx / chronic PID | 7/626 (1.1) | 0/61 (0.0) |  | 5/552 (0.9) | 0/79 (0.0) |
| Peritoneal pseudocyst | 6/626 (1.0) | 0/61 (0.0) |  | 0/552 (0.0) | 0/79 (0.0) |
| Abscess / salpingitis / PID | 0/626 (0.0) | 0/61 (0.0) |  | 5/552 (0.9) | 0/79 (0.0) |
| Fibroma / fibrothecoma / thecofibroma | 5/626 (0.8) | 0/61 (0.0) |  | 1/552 (0.2) | 0/79 (0.0) |
| Benign rare tumor | 3/626 (0.5) | 0/61 (0.0) |  | 0/552 (0.0) | 0/79 (0.0) |
| Hydrosalpinx or cystadenoma | 1/626 (0.2) | 0/61 (0.0) |  | 0/552 (0.0) | 0/79 (0.0) |
| Borderline tumor | 0/626 (0.0) | 38/61 (62.3) |  | 0/552 (0.0) | 56/79 (70.9) |
| Primary invasive tumor | 0/626 (0.0) | 11/61 (18.0) |  | 0/552 (0.0) | 13/79 (16.5) |
| Metastases to the ovary | 0/626 (0.0) | 2/61 (3.3) |  | 0/552 (0.0) | 3/79 (3.8) |
| Not possible | 18/626 (2.9) | 7/61 (11.5) |  | 30/552 (5.4) | 4/79 (5.1) |
| Not performed (IOTA1b) | 23/626 (3.7) | 3/61 (4.9) |  | 34/552 (6.2) | 3/79 (3.8) |
| Results are given as n/N (%) for categorical variables and as median (min-max) for continuous characteristics. PID: Pelvic Inflammatory Disease. | | | | | |
